# The Fight against HIV-Associated Disseminated Histoplasmosis in the Americas: Unfolding the Different Stories of Four Centers

**DOI:** 10.3390/jof5020051

**Published:** 2019-06-17

**Authors:** Mathieu Nacher, Terezinha Silva Leitao, Beatriz L. Gómez, Pierre Couppié, Antoine Adenis, Lisandra Damasceno, Magalie Demar, Blanca Samayoa, Diego H. Cáceres, Roger Pradinaud, Anastacio de Queiroz Sousa, Eduardo Arathoon, Angela Restrepo

**Affiliations:** 1 Centre d’Investigation Clinique Antilles Guyane, Inserm CIC1424, Centre Hospitalier de Cayenne, Cayenne, French Guiana; 2 EA3593 Ecosystèmes Amazoniens et Pathologie Tropicale (EPaT), Université de Guyane, DFR Santé, 97300 Cayenne, French Guiana; pierre.couppie@ch-cayenne.fr (P.C.); magalie.demar@ch-cayenne.fr (M.D.); 3 Departamento de Doenças Infecciosas, Universidade Federal do Ceará, Fortaleza, 60020-181 Ceará, Brasil; tsilva@ufc.br (T.S.L.); lisandraserra@yahoo.com.br (L.D.); aqsousa@gmail.com (A.d.Q.S.); 4 Studies in Translational Microbiology and Emerging Diseases (MICROS) Research Group, School of Medicine and Health Sciences, Universidad del Rosario, 110111 Bogota, Colombia; beatrizlgomez@hotmail.com (B.L.G.); diegocaceres84@gmail.com (D.H.C.); 5 Service de Dermatologie Vénéréologie, Centre Hospitalier de Cayenne, 97300 Cayenne, French Guiana; rogerpradinaud@orange.fr; 6 Centre d'Investigation Clinique Epidémiologie Clinique Antilles Guyane CIC INSERM 1424, Cayenne General Hospital, Cayenne, French Guiana; antoine.adenis@ch-cayenne.fr; 7 Laboratoire de Parasitologie-Mycologie, Centre Hospitalier de Cayenne, 97300 Cayenne, French Guiana; 8 Asociación de Salud Integral & Hospital General San Juan de Dios, 01001 Guatemala City, Guatemala, blanca.samayoa@gmail.com; 9 La Clinica Familiar de Luis Angel Garcia, 01001 Guatemala City, Guatemala; earathoon@hotmail.com; 10 Corporacion para Investigaciones Biologicas, Hospital Pablo Tobon Uribe, 11001 Medellin, Colombia; angelares@une.net.co

**Keywords:** histoplasmosis, awareness, HIV, Latin America, leishmaniasis

## Abstract

Disseminated histoplasmosis is a major opportunistic infection of HIV-infected patients, killing thousands in Latin America each year. Yet, it remains a neglected disease that is often confused with tuberculosis, for lack of simple, affordable, and rapid diagnostic tools. There is great heterogeneity in the level of histoplasmosis awareness. The purpose of this report was to describe how the historical “awakening” to the threat of histoplasmosis came to be in four different centers that have actively described this disease: In Brazil, the Sao José hospital in Fortaleza; in Colombia, the Corporación para Investigaciones Biológicas in Medellin; in French Guiana, Cayenne Hospital; and in Guatemala, the Association de Salud Integral in Guatemala city. In Brazil and French Guiana, the search for leishmaniasis on the buffy coat or skin smears, respectively, led to the rapid realization that HIV patients were suffering from disseminated histoplasmosis. With time and progress in fungal culture, the magnitude of this problem turned it into a local priority. In Colombia and Guatemala, the story is different because for these mycology centers, it was no surprise to find histoplasmosis in HIV patients. In addition, collaborations with the CDC to evaluate antigen-detection tests resulted in researchers and clinicians developing the capacity to rapidly screen most patients and to demonstrate the very high burden of disease in these countries. While the lack of awareness is still a major problem, it is instructive to review the ways through which different centers became histoplasmosis-aware. Nevertheless, as new rapid diagnostic tools are becoming available, their implementation throughout Latin America should rapidly raise the level of awareness in order to reduce the burden of histoplasmosis deaths.

Disseminated histoplasmosis is a neglected killer in the Americas [1]. This has been overlooked by many National AIDS programmes and international AIDS authorities [2]. Scarce publications from cohorts of HIV patients show that some teams of clinicians and mycologists, scattered throughout South and Central America, have come to the same conclusion [3,4,5,6]. The shared sense of a global tragedy that has been unfolding in South and Central America naturally arose through visits in numerous hospitals were patients where obviously dying without a diagnosis.

However, upon closer attention, the histories of how these hospitals started to realize that histoplasmosis was killing many of their HIV-infected patients differ in interesting ways. We here narrate how awareness unfolded during the time span of the HIV epidemic. 

French Guiana is a sparsely populated French overseas territory. Cutaneous leishmaniasis is a common disease in persons in contact with the forest. Dermatologists at Cayenne hospital thus have always had microscopes in consultation rooms so that they can read skin smears. After leishmaniasis, they began making smears of other types of lesions, looking for scabies, herpes, etc. When the HIV epidemic started, dermatologists who were already dealing with other sexually transmitted diseases, became the main care providers for HIV patients. In the 1980s, among the first AIDS cases, some patients came with cutaneous or mucous lesions, and they benefitted from a smear, which demonstrated the presence of *Histoplasma capsulatum*. Although publications in the 1950s at the Pasteur Institute in Cayenne had described the presence of *H. capsulatum* in French Guiana, it was the dermatologists’ proactive search for pathogens in skin lesions that made the diagnoses, thus shedding light on the problem. However, these patients often had very advanced stages of disseminated histoplasmosis, and some were often wrongly diagnosed with cachectic syndromes or culture-negative tuberculosis. Meanwhile, the HIV epidemic progressed, with over 1% of pregnant women being HIV-positive. In 1997, a mycologist arrived at the recently created university laboratory at Cayenne Hospital and she started a fungal culture. This revealed a far greater number of histoplasmosis cases than previously thought (Figure 1a). Dermatologists carefully compiled clinical observations, and then analyzed them to gain a better knowledge of this disease. Soon, every new resident was briefed on histoplasmosis, and any suggestion of a possible case of histoplasmosis led to increasingly aggressive paraclinical investigations (such as bone marrow aspiration or tissue biopsy (liver, lymph node, digestive mucosae)), with a policy of presumptive treatment. The small hospital allowed for synergy between mycologists and clinicians, further educating surgeons and endoscopists to keep some biopsy samples out of formalin for culture. Soon, this knowledge spread to the other two hospitals of French Guiana, and HIV-associated histoplasmosis was found everywhere and mortality of the diseased dropped [7]. As a clinical investigation center was created, this allowed researchers to further analyze the data to learn more about the disease and to raise funds for research projects of greater complexity. The clinical questions and the laboratory questions were now enriched by an epidemiological and global health perspective. This three-phased 30-year assemblage of different competencies led to knowledge and proactive philosophy in treating patients with histoplasmosis that greatly helped reduce mortality despite the lack of an antigen-detection technique [7]. The comparison of practices and the exchange of experiences with neighboring countries led to the realization that the trajectory taken in French Guiana was made possible by a convergence of favorable conditions, catalyzed by the small size of the hospital, allowing different specialties and their inputs to optimize patient care.

In Fortaleza, Brazil, in the early 1980s, visceral leishmaniasis was already a well-known endemic disease, and histoplasmosis was considered a rare event. Coincidentally with the beginning of the AIDS epidemic in Fortaleza, the first cases of disseminated histoplasmosis (DH) were “accidentally” diagnosed when buffy coat was used to look for *Leishmania* in the peripheral blood of patients with presentations compatible with Kala-azar. A microbiologist (Jacó Ricarte L. Mesquita) working at the laboratory of São José Hospital, increasingly saw images of yeasts inside leukocytes in peripheral blood smears of febrile AIDS patients. He sent these samples for fungal culture at a reference laboratory at the Federal University of Ceará, which identified the presence of *Histoplasma*. He then decided to apply the method to concentrate the leukocytes (buffy coat) to help identify *Histoplasma* through direct examination and culture (Figure 2). This finding incited clinicians to use buffy coat direct examinations routinely in that area of Brazil to diagnose DH, because it was easy to perform, cheap, and could promote rapid decision-making. However, given the low sensitivity of this test, the laboratory of the São José hospital, the main reference for AIDS treatment in the state, started systematic cultures of the buffy coat. This led to an increased number of cases diagnosed. However, the mortality due to DH in the late 1990s was still high (65%), and death occurred on an average 7.2 days after hospital admission. Subsequently, clinicians increased their expertise on the disease and conducted important studies to show the burden of histoplasmosis, and further adapted patient care to this knowledge [3,8]. After the inclusion of lactate dehydrogenase as a screening test for DH in AIDS patients with fever, since the late 1990s, more presumptive diagnoses were made and mortality decreased to around 40%, which is still high and probably reflects late diagnoses in the absence of novel methods to diagnose *Histoplasma* in Fortaleza. Here, the primary catalysts for the awareness and improved treatment of DH were the investigation strategies in an endemic area for Kala-Azar, but the trajectory taken in Fortaleza was also favored by a convergence of favorable conditions, catalyzed by the small size of the hospital allowing for a virtuous cycle to take place once it became clear that histoplasmosis was there.

Colombia is a strategically located country that connects Central and South America. In the 1960s and with the support of the School of Medicine, University of Antioquia, the first diagnostic service in medical mycology became established. Shortly thereafter, the first reported studies describing cases of histoplasmosis and the methods for its diagnosis by means of serology and histopathology were published. Studies on the skin-test reactivity to histoplasmin were carried out during the late 1960s and early 1970s, demonstrating an overall reactivity of 21% in healthy individuals residing in five different regions of the country. Years later (1978), a new medical mycology unit in charge of diagnosis and clinical research was founded in Medellín, at the Corporación para Investigaciones Biológicas (CIB). The initial clinical and therapeutic trials began in the late 1970s, through multicentric studies designed to evaluate different antifungal compounds. In the 1980s, histoplasmosis diagnoses were carried out by conventional methods: Stains, histopathology, culture, and serological tests (immunodiffusion and complement fixation). Around the same time, the HIV epidemic began in Colombia, leading to a significant increase of patients with progressive disseminated histoplasmosis (PDH) exhibiting a high mortality. Research in the mid-1990s then focused towards the development of new diagnostic methods, this time with the novel approach of detecting circulating antigens using monoclonal antibodies. Epidemiological studies were also designed to collect and analyze demographic data, risk factors, and patients’ clinical manifestations, in collaboration with the Colombian National Institute of Health (Instituto National de Salud, Bogota, Colombia). The first reports of Colombian histoplasmosis outbreaks appeared at about the same time. Histoplasmosis has thus been well known long before the arrival of the HIV epidemic. However, since histoplasmosis is not a reportable disease in Colombia, epidemiological data are scarce and the real burden of the disease is still unclear.

From 2005 until now, with the expertise and support of other groups and colleagues, interests have been focused on the development and validation of molecular tests, and the implementation of new antigen-detection methods. Work has also focused on describing well-characterized cohorts of HIV patients with histoplasmosis and determining the particular role of co-infections with tuberculosis, which are present in approximately 50% of cases and hinder prompt diagnosis and treatment. During all these years, emphasis was placed on training medical and laboratory personnel so that they may suspect histoplasmosis and use appropriate diagnostic procedures and treatments. This strategy resulted in an increase in diagnosis, with a doubling of the number of cases per year and a reduction of mortality. Overall, in Colombia, the mycological knowledge was there when the HIV epidemic started, and it has percolated towards HIV clinicians through proactive training of clinicians and the development of diagnostic tools (Figure 1b). Although preexisting mycological expertise was the first catalyst to becoming aware of the importance of histoplasmosis*,* the trajectory taken in Colombia, as in French Guiana, was favored by a convergence of favorable conditions, catalyzed by the proximity and small size of the hospital allowing different specialties and their inputs to optimize patient care. 

In Guatemala, the association Asociación de Salud Integral and Hospital General San Juan de Dios combine the mycological expertise and clinical expertise of a clinician mycologist, who is very well aware of the omnipresence of fungal pathogens in Latin America. However, direct examination and culture were often insufficiently sensitive to diagnose patients early enough. A collaboration with the CDC’s mycotic branch led to the validation of a histoplasma antigen Enzyme Immuno Assay that considerably increased the number of histoplasmosis cases detected and helped reduce mortality [9,10,11]. Thus, although awareness was always there, the arrival of a new rapid and sensitive diagnostic tool allowed researchers and clinicians to reach new levels of awareness: Histoplasmosis is the first killer of HIV patients in the first year following diagnosis. In Guatemala, although for mycologists the world was always full of fungi, the experience has been that the more you look for histoplasmosis, the more you find it. This permanent effort to diagnose histoplasmosis was facilitated by rapid tests and has led to significant reduction of mortality. For mycologists in Guatemala, it was not the presence of histoplasmosis that was surprising, but the magnitude of its burden.

Diagnosis of histoplasmosis by antigen test detection has been available since 1986 by a private laboratory in the USA (MiraVista). No commercially available test existed for use by other countries, and sending samples to the USA was difficult and expensive, and thus testing was never done. Very recently, a commercial ELISA for antigen detection was made available (Histoplasma Galactomannan EIA, IMMY™, USA), and in 2018, this kit was evaluated in a multicenter validation by Colombia and Guatemala. This commercial antigen test showed a high performance and reproducibility, suggesting that it can be used to detect progressive disseminated histoplasmosis in persons living with HIV in countries where histoplasmosis is highly endemic. This finding has facilitated the implementation of the technology in other Latin American countries, including Panama, Honduras, Nicaragua, Costa Rica, Peru, Argentina, Brazil, and Chile.

Hence, different teams working in very different conditions in South and Central America have come to the same conclusions: Histoplasmosis is killing their HIV patients [12,13,14]. This awareness, and the efforts to diagnose and treat histoplasmosis early have allowed them to roll back HIV mortality. However, these stories are unfortunately the exception rather than the rule, and there are numerous territories in South and Central America where histoplasmosis is still confused with tuberculosis, with fatal consequences.

## Figures and Tables

**Figure 1:**
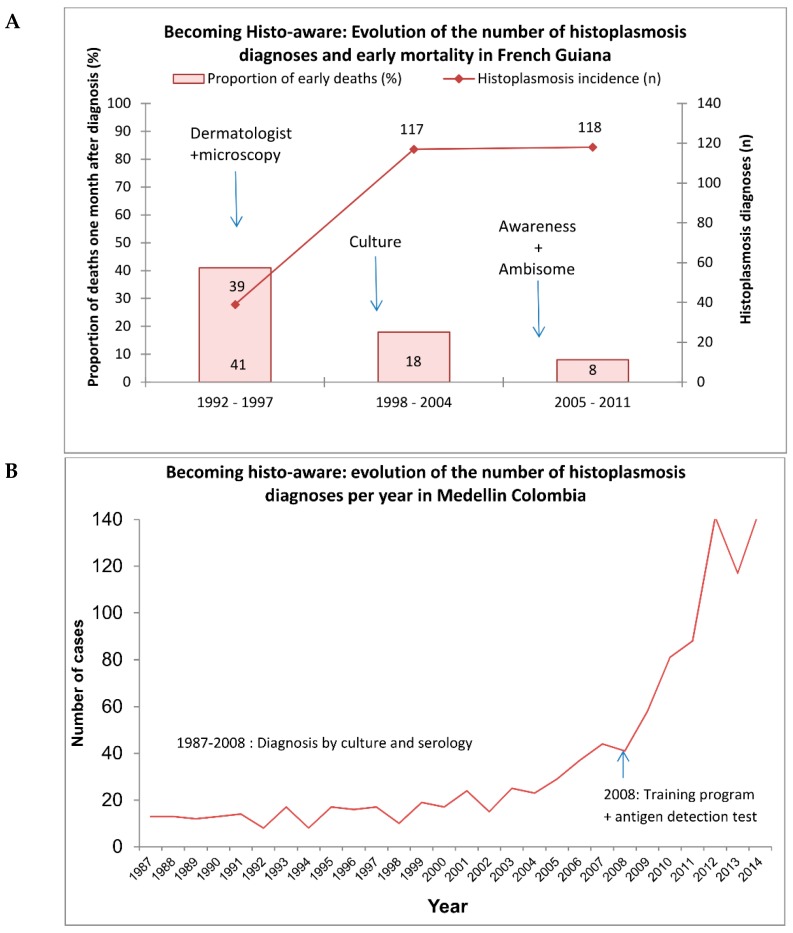
Becoming histoplasmosis-aware (“histo-aware”) in (**A**) French Guiana and (**B**) Colombia.

**Figure 2:**
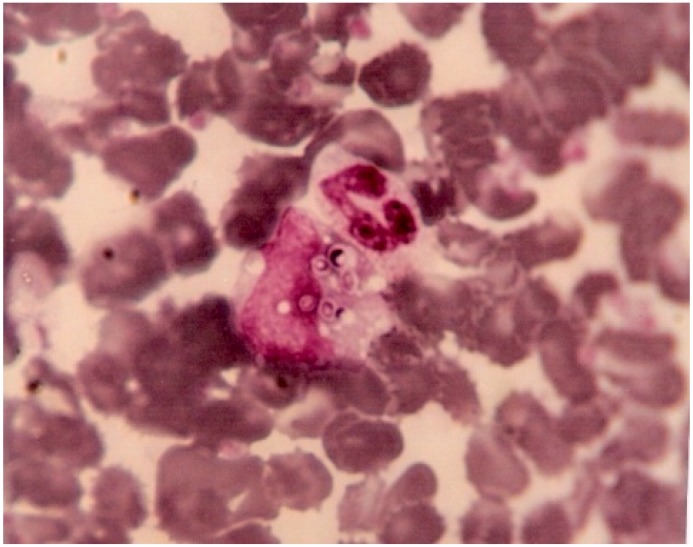
*Histoplasma capsulatum* yeasts on a stained buffy coat (Giemsa ×100), Ceara, Brazil.

## References

[1] Nacher M., Adenis A., Mc Donald S., Do Socorro Mendonca Gomes M., Singh S., Lopes Lima I., Malcher Leite R., Hermelijn S., Wongsokarijo M., Van Eer M. (2013). Disseminated Histoplasmosis in HIV-Infected Patients in South America: A Neglected Killer Continues on Its Rampage. PLoS Negl. Trop. Dis..

[2] Neglected Histoplasmosis in Latin America Group (2016). Disseminated histoplasmosis in Central and South America, the invisible elephant: The lethal blind spot of international health organizations. AIDS.

[3] Daher E.F., Silva G.B., Barros F.A., Takeda C.F., Mota R.M., Ferreira M.T., Oliveira S.A., Martins J.C., Araújo S.M.H.A., Gutiérrez-Adrianzén O.A. (2007). Clinical and laboratory features of disseminated histoplasmosis in HIV patients from Brazil. Trop. Med. Int. Health..

[4] Caceres D.H., Zuluaga A., Arango-Bustamante K., de Bedout C., Tobon AM., Restrepo A., Gómez B.L., Cano L.E., González Á. (2015). Implementation of a Training Course Increased the Diagnosis of Histoplasmosis in Colombia. Am. J. Trop. Med. Hyg..

[5] Nacher M., Adenis A., Adriouch L., Dufour J., Papot E., Hanf M., Vantilcke V., Calvez M., Aznar C., Carme B. (2011). What is AIDS in the Amazon and the Guianas? Establishing the burden of disseminated histoplasmosis. Am. J. Trop. Med. Hyg..

[6] Adenis A.A., Valdes A., Cropet C., McCotter O.Z., Derado G., Couppie P., Chiller T., Nacher M. (2018). Burden of HIV-associated histoplasmosis compared with tuberculosis in Latin America: A modelling study. Lancet Infect. Dis..

[7] Adenis A., Nacher M., Hanf M., Vantilcke V., Boukhari R., Blachet D., Demar M., Aznar C., Carme B., Couppie P. (2014). HIV-associated histoplasmosis early mortality and incidence trends: From neglect to priority. PLoS Negl. Trop. Dis..

[8] Medina N., Samayoa B., Lau-Bonilla D., Denning D.W., Herrera R., Mercado D., Guzmán B., Pérez J., Arathoon E. (2017). Burden of serious fungal infections in Guatemala. Eur. J. Clin. Microbiol. Infect. Dis..

[9] Dasmasceno LS., Novaes A.R., Alencar C.H.M., Lima D.T., Sidrim J.J.C., Gonçalves M.V.F., de Mesquita J.R.L., Leitão T.D.M.J.S. (2013). Disseminated histoplasmosis and aids: Relapse and late mortality in endemic area in north-eastern Brazil. Mycoses.

[10] Samayoa B., Roy M., Cleveland A.A., Medina N., Lau-Bonilla D., Scheel C.M., Gomez B.L., Chiller T., Arathoon E. (2017). High Mortality and Coinfection in a Prospective Cohort of Human Immunodeficiency Virus/Acquired Immune Deficiency Syndrome Patients with Histoplasmosis in Guatemala. Am. J. Trop. Med. Hyg..

[11] Scheel C.M., Samayoa B., Herrera A., Lindsley M.D., Benjamin L., Reed Y., Hart J., Lima S., Rivera B.E., Raxcaco G. (2009). Development and Evaluation of an Enzyme-Linked Immunosorbent Assay to Detect Histoplasma capsulatum Antigenuria in Immunocompromised Patients. Clin. Vaccine Immunol..

[12] Gutierrez M.E., Canton A., Sosa N., Puga E., Talavera L. (2005). Disseminated histoplasmosis in patients with AIDS in Panama: A review of 104 cases. Clin. Infect. Dis..

[13] Assi M.A., Sandid M.S., Baddour L.M., Roberts G.D., Walker R.C. (2007). Systemic histoplasmosis: A 15-year retrospective institutional review of 111 patients. Med. Baltim..

[14] Pietrobon D., Negro-Marquinez L., Kilstein J., Galindez J., Greca A., Battagliotti C. (2004). Disseminated histoplasmosis and AIDS in an Argentine hospital: Clinical manifestations, diagnosis and treatment. Enferm. Infecct. Microbiol. Clin..

